# Graves' Thyrotoxicosis Leading to Adrenal Decompensation and Hyperandrogenemia in a Pediatric Patient with Salt-Wasting Congenital Adrenal Hyperplasia

**DOI:** 10.1155/2018/2359205

**Published:** 2018-11-22

**Authors:** Meghan E. Fredette, Lisa Swartz Topor

**Affiliations:** Pediatric Endocrinology, Rhode Island Hospital/The Warren Alpert Medical School of Brown University, 111 Plain Street, 3rd Floor, Providence, RI 02903, USA

## Abstract

**Introduction:**

Thyroid hormone is known to accelerate glucocorticoid turnover. In a thyrotoxic state, individuals with adrenal insufficiency are unable to increase endogenous cortisol production to compensate for increased turnover, placing them at risk for symptoms of glucocorticoid deficiency and adrenal crisis. In patients with salt-wasting congenital adrenal hyperplasia (SW-CAH), hyperandrogenemia is a measurable reflection of relative glucocorticoid insufficiency.

**Case Presentation:**

A 12-year-old girl with SW-CAH reported 3 recent episodes of vomiting without diarrhea, and accompanying tachycardia, responsive to stress dose steroids. In the previous 9 months, she unintentionally lost 2.6 kg. She had tachycardia and new thyromegaly. Labs showed suppressed TSH, elevated free T4 and total T3, and elevated thyroid stimulating immunoglobulin (TSI) consistent with Graves' disease. Adrenal androgens were markedly elevated. Maintenance hydrocortisone dose was 25 mg/m^2^/day and was not changed. Methimazole was initiated. Four weeks later, free T4 and adrenal androgens normalized. She had no further vomiting episodes.

**Conclusions:**

Thyrotoxicosis must be included in the differential diagnosis of individuals with SW-CAH who present with episodes concerning for adrenal crises, escalating hydrocortisone requirements, and/or inadequate suppression of adrenal hormones.

## 1. Introduction

Salt-wasting congenital adrenal hyperplasia (SW-CAH) is the most common cause of primary adrenal insufficiency in pediatric patients, and in over 95% of cases it is caused by a mutation in the* CYP21A2 *gene, which causes a deficiency of the enzyme 21-hydroxylase [1]. Deficiency in 21-hydroxylase causes both gluco- and mineralocorticoid deficiency as well as hyperandrogenism due to the diversion of adrenal steroid precursors to adrenal androgens. Treatment consists of mineralocorticoid supplementation, as well as supraphysiologic glucocorticoid doses to suppress ACTH and minimize formation of adrenal androgens.

Management of glucocorticoid dosing in the growing child with SW-CAH is challenging. Typical dosing of hydrocortisone ranges from 10 to 15 mg/m^2^ daily in 3 divided doses [1]. Cortisol clearance increases in puberty, and glucocorticoid dose requirements often increase [2]. Glucocorticoid overtreatment can compromise height and result in symptoms of glucocorticoid excess. Undertreatment can also compromise height due to accelerated epiphyseal maturation and can result in bothersome virilization and prompt adrenal crises. Close monitoring of growth and biochemical measurement of androgens are essential for the care of the pediatric patient with SW-CAH.

In this case report, we describe a 12-year-old female with previously well-controlled SW-CAH, who presented to a pediatric emergency department with multiple episodes concerning for adrenal crises, responsive to stress dose steroids. She was found to have poorly suppressed androgens on supraphysiologic glucocorticoid doses. Ultimately, she was diagnosed with Graves' disease.

## 2. Case Presentation

A 12-year-old female with SW-CAH presented to the pediatric endocrinology clinic for routine follow-up. She was diagnosed with SW-CAH in the newborn period after presenting with ambiguous genitalia and was treated with supraphysiologic hydrocortisone divided three times daily throughout her life (prepubertal dosing range 10-15 mg/m^2^/day, pubertal dosing range 15-25 mg/m^2^/day), as well as fludrocortisone (0.1 mg daily). She was monitored every 3-4 months with clinical examinations, growth parameters, and serum measurements of 17-hydroxyprogesterone (17-OHP), androstenedione, and testosterone (Esoterix Laboratory, Calabasas Hills, CA), which together guided her medication dosing. She had no evidence of glucocorticoid excess and her growth velocity was normal, without evidence of acceleration or suppression. She and her parents reported continued excellent compliance with her medication regimen.

**Figure 1:**
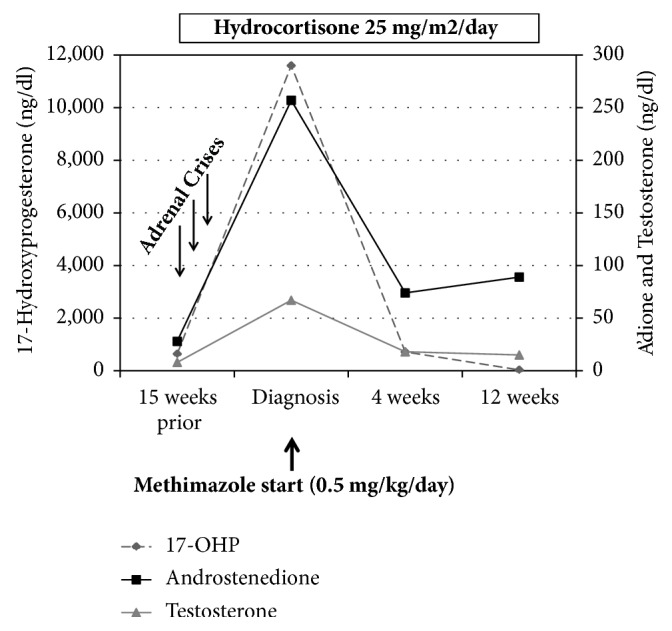
Graphic demonstration of adrenal androgen levels over time.

At age 12 3/12 years, she reported 3 recent emergency department visits for persistent vomiting without diarrhea, abdominal pain, inability to tolerate oral steroids, and tachycardia. One of the episodes was accompanied by fever >39°C. There was no hypotension during any of these episodes, but heart rates were elevated ranging from 124 to 154 beats per minute. Sodium and potassium were normal: Na 137-140 meq/L (reference range, 133-143 meq/L) and K 3.6-4.1 meq/L (reference range 3.4-4.7 meq/L). Each episode was treated with normal saline boluses and intravenous stress dose steroids, and treatment led to immediate improvement in symptoms. She was discharged from the emergency department with recommendations for stress dose steroids by mouth. Over the prior 9 months, she had also unintentionally lost 2.6 kg and had an accelerated annualized growth velocity of 10.6 centimeters per year. BMI was 15.4 kg/m^2^ (Z-score -1.38). She denied any heat intolerance, jitteriness, palpitations, sweating, diarrhea, or vision complaints. While in clinic, her resting pulse was elevated at 136 beats per minute and blood pressure was normal. She was noted to have new symmetric thyromegaly without nodules. She had no lid lag or stare. She was in mid-puberty, with tanner 3 breasts. There was no family history of autoimmune disease or thyroid disease. Labs (Table 1) showed a suppressed TSH, and an elevated free T4 and Total T3. Thyroid antibodies were consistent with Graves' disease, with a thyroid stimulating immunoglobulin (TSI) of 652% (reference range <140%). She had no prior thyroid studies for comparison. Adrenal androgens were markedly elevated with 17-OHP 11,600 ng/dl (reference range 11-155 ng/dl, target <1000 ng/dl in SW-CAH). At a clinic visit 15 weeks prior to this evaluation, her 17-OHP on the same dose of hydrocortisone (25 mg/m^2^/day) was improved at 639 ng/dl. She was initiated on methimazole 0.5 mg/kg/day. Her hydrocortisone dose was not changed and remained at 25 mg/m^2^/day. Four weeks later, repeat labs demonstrated normal free T4 with mildly suppressed TSH and normal total T3. 17-OHP was stable at 717 ng/dl (Figure 1). She had no further vomiting episodes after initiation of methimazole, and her tachycardia resolved.

Three months after treatment began for Graves' disease, her TSH and free T4 were in the reference range, and methimazole was decreased to 0.3 mg/kg/day. Repeat 17-OHP was low at 33 ng/dl, indicating suppression on the unchanged hydrocortisone dose of 25 mg/m^2^/day. Her hydrocortisone dose was decreased to 21 mg/m^2^/day.

## 3. Discussion

Thyroid hormone is well-known to accelerate the metabolism of cortisol. In the adrenally sufficient individual, the thyrotoxic state both increases the production of cortisol, and shortens the half-life of cortisol due to an increased turnover rate, with a net effect of normal circulating cortisol levels [3]. Cortisol requirements are increased due to the stress and increased metabolic demands of thyrotoxicosis. The mechanism of increased cortisol clearance appears to be due to thyroid hormone effects on the activity of 11*β*HSD and 5-*α* reductase enzymes [4].

Patients with adrenal insufficiency are unable to mount an adrenal response by increasing cortisol levels in the setting of excess thyroid hormone, placing them at risk for adrenal crises. Recurrent adrenal crises have been reported in individuals with autoimmune primary adrenal insufficiency upon development of autoimmune thyrotoxicosis [5]. Similarly, it is well documented that hypothyroid patients initiated on thyroid hormone repletion with undiagnosed primary or secondary adrenal insufficiency can present with adrenal crisis [6, 7]. In the hypothyroid state, cortisol clearance is slowed, protecting the hypothyroid, adrenally insufficient patient from symptoms of adrenal insufficiency. With thyroid hormone initiation, cortisol clearance increases, and adrenal crisis may occur, similar to the thyrotoxic patient with adrenal insufficiency.

The literature includes only 2 cases of adult patients with congenital adrenal hyperplasia who experienced symptomatic adrenal insufficiency and/or adrenal crisis associated with thyrotoxicosis. Takasu et al. described a 75-year-old female who was admitted for treatment of Graves' thyrotoxicosis and was found unconscious due to adrenal crisis during her hospitalization [8]. She was ultimately diagnosed with nonclassical 21-hydroxylase deficiency. Kim et al. reported the case of a 23-year-old male with Graves' thyrotoxicosis who was diagnosed with nonclassic 11-beta hydroxylase deficiency after he presented with hypokalemia and hypertension and was found to have adrenal hyperplasia on imaging [9]. Our report is the first case describing a pediatric patient with CAH, who experienced repeated episodes concerning for adrenal crises and hyperandrogenemia secondary to the onset of Graves' thyrotoxicosis, demonstrating the impact of thyroid hormone excess on the metabolism of glucocorticoids* in vivo*.

## 4. Conclusions

This unusual case demonstrates the unique findings of thyrotoxicosis in a pediatric patient with SW-CAH on glucocorticoid supplementation. She presented with classic signs and symptoms of Graves' disease including thyromegaly, weight loss, and tachycardia, as well as SW-CAH specific symptoms reflecting cortisol deficiency including biochemical hyperandrogenemia and repeated episodes concerning for adrenal crises. Increased clearance of cortisol in the setting of thyrotoxicosis resulted in recurrent adrenal decompensations due to insufficient cortisol, though 1 episode was accompanied by fever and thus may have been triggered by illness. Hyperandrogenemia and episodes concerning for adrenal crises resolved with normalization of thyroid levels, without any change in glucocorticoid dose. Thyrotoxicosis must be included in the differential diagnosis of individuals with any form of adrenal insufficiency, including CAH, who present with escalating glucocorticoid requirements, or recurrent adrenal crises despite appropriate glucocorticoid adherence and dosing.

## Figures and Tables

**Table 1:** Androgens and thyroid studies at baseline, diagnosis, and after 4 and 12 weeks of treatment.

**Lab Value**	**Reference Range**	**15 Weeks prior**	**Diagnosis**	**4 weeks**	**12 weeks**
TSH (uIU/ml)	0.35-5.5		0.019	0.021	4.345
Total T3 (ng/dl)	82-213		331	150	99
Free T4 (ng/dl)	0.8-1.8		2.82	1.42	0.89
17-Hydroxyprogesterone (ng/dl)	11-155	639	11,600	717	33
Androstenedione (ng/dl)	50-170	28	257	74	89
Testosterone (ng/dl)	15-35	8	67	18	15
